# High serum levels of pregenomic RNA reflect frequently failing reverse transcription in hepatitis B virus particles

**DOI:** 10.1186/s12985-018-0994-7

**Published:** 2018-05-15

**Authors:** Kasthuri Prakash, Gustaf E. Rydell, Simon B. Larsson, Maria Andersson, Gunnar Norkrans, Heléne Norder, Magnus Lindh

**Affiliations:** Department of Infectious Diseases, Sahlgrenska Academy, University of Gothenburg, Guldhedsgatan 10B, 413 46 Gothenburg, Sweden

## Abstract

**Background:**

Hepatocytes infected by hepatitis B virus (HBV) produce different HBV RNA species, including pregenomic RNA (pgRNA), which is reverse transcribed during replication. Particles containing HBV RNA are present in serum of infected individuals, and quantification of this HBV RNA could be clinically useful.

**Methods:**

In a retrospective study of 95 patients with chronic HBV infection, we characterised HBV RNA in serum in terms of concentration, particle association and sequence. HBV RNA was detected by real-time PCR at levels almost as high as HBV DNA.

**Results:**

The HBV RNA was protected from RNase and it was found in particles of similar density as particles containing HBV DNA after fractionation on a Nycodenz gradient. Sequencing the epsilon region of the RNA did not reveal mutations that would preclude its binding to the viral polymerase before encapsidation. Specific quantification of precore RNA and pgRNA by digital PCR showed almost seven times lower ratio of precore RNA/pgRNA in serum than in liver tissue, which corresponds to poorer encapsidation of this RNA as compared with pgRNA. The serum ratio between HBV DNA and HBV RNA was higher in genotype D as compared with other genotypes.

**Conclusions:**

The results suggest that HBV RNA in serum is present in viral particles with failing reverse transcription activity, which are produced at almost as high rates as viral particles containing DNA. The results encourage further studies of the mechanisms by which these particles are produced, the impact of genotype, and the potential clinical utility of quantifying HBV RNA in serum.

**Electronic supplementary material:**

The online version of this article (10.1186/s12985-018-0994-7) contains supplementary material, which is available to authorized users.

## Background

Chronic infection with hepatitis B virus (HBV) may induce inflammation and lead to liver cirrhosis and hepatocellular carcinoma (HCC) [1]. The risk of developing these complications is greater if a high degree of viral replication persists beyond the age of 30–40 years. Accordingly, quantification of HBV DNA in serum is the main marker for predicting the risk of cirrhosis and HCC [2], and HBV DNA levels are also measured to monitor the response to antiviral treatment [1]. During nucleoside analogue treatment, HBV DNA usually soon declines to low or undetectable levels in serum. After that, quantification of HBV DNA gives no indication of whether the treatment reduces the number of infected hepatocytes or number of covalently closed circular (cccDNA) copies in these cells, and therefore quantification of hepatitis B surface antigen (HBsAg) [3–5] and recently HBV RNA [6, 7] have emerged as adjunctive markers for on-going HBV replication.

The cccDNA episome of HBV in the nucleus of infected hepatocytes is transcribed to several mRNAs during the viral replication. One of these transcripts, the pregenomic RNA (pgRNA), is reverse transcribed to HBV DNA by the viral polymerase during the formation of new viral capsids in the cytoplasm of the hepatocytes before release of the enveloped virions, but HBV RNA containing particles can also be detected in serum [8]. A potential diagnostic value of quantifying HBV RNA in serum was suggested in a few studies from Japan 10 years ago [9], but was not established in clinical diagnostics. A clinical interest for this analysis has been revived by two recent studies, which proposed HBV RNA quantification as a complement to HBV DNA to monitor therapeutic effects [6, 7]. Because nucleoside analogues act on reverse transcription, the HBV RNA levels should not be directly influenced by the drug, but a decline of HBV RNA should rather represent indirect effect of treatment, including effects on the amount of cccDNA or the rate of transcription. The results from the mentioned studies reported that HBV RNA in serum was contained in virus-like particles, that serum levels of HBV RNA and HBV DNA correlated, and that levels of HBV RNA declined less than HBV DNA during antiviral treatment, but significantly more than HBsAg levels.

In the present study we compared HBV RNA and DNA levels in 95 patients with chronic HBV infection, and performed targeted analyses to further characterise the HBV RNA.

## Methods

### Patients and samples

This is a retrospective analysis of stored samples from a previous study [10], which included 160 patients with chronic HBV infection. The present study includes 95 patients (32 HBeAg-positive, 63 HBeAg-negative) from whom stored samples were available and whose HBV strains in previous testing have been classified as genotype A, B, C or D.

### Extraction of HBV nucleic acids from serum

Nucleic acids were extracted in a MagNA Pure LC instrument (Roche Applied Science) using the Total NA protocol and 200 μL of serum as input volume and 100 μL elusion volume.

### Quantification of HBV DNA and HBV RNA in serum

A real-time PCR assay targeting a conserved segment of the core region was used to quantify HBV DNA and HBV RNA in parallel. Amplifications were performed in an ABI Quantstudio 6 instrument, using forward primer, GGTCCCCTAGAAGAAGAACTCCCT (nt 2367–2390), reverse primer, CATTGAGATTCCCGAGATTGAGAT (nt 2454–2431), and probe, TCTCAATCGCCGCGTCGCAGA (nt 2408–2428). DNA and RNA amplification was carried out in 25 μL reaction mixtures containing 12.5 μL TaqMan Universal PCR master mix (Applied Biosystems, CA, USA) for DNA and 12.5 μL SuperScript III Platinum One-Step qRT PCR kit with ROX (Invitrogen) for RNA, 0.2 μM of each primer and probe, and 5 μL of extracted nucleic acids. Initial UNG and RT (48°C, 30 min) and denaturation steps (95°C, 10 min) were followed by 45 cycles of amplification (95°C, 15 s; 58°C, 1 min). All measurements were carried out in duplicates.

Quantification of HBV RNA was preceded by a TURBO DNase (Thermo Fisher Scientific) incubation step according to the instructions from the manufacturer). In parallel with the one-step RT-PCR, the DNase treated samples were also run in the real-time PCR with a DNA master mix (without reverse transcriptase and without an RT step) to confirm that DNA degradation had been effective and that the RT-PCR did not amplify undigested HBV DNA.

### Characterisation of HBV RNA particles by RNase treatment and fractionation

In order to study if HBV RNA was encapsidated, one serum sample with a high HBV RNA level was reanalysed with or without an RNase incubation step prior to MagNA Pure extraction. To characterise the particles that contained HBV RNA we centrifuged serum in order to identify the type of particles that contained HBV RNA. First, 1.5 mL of each of six concentrations of Nycodenz (50, 42, 33, 25, 16 and 8 wt%) in Williams medium was added to two thinwall Ultra-Clear 17 mL ultracentrifuge tubes (Beckman Coulter), with care not to disturb interfaces. Then, two tubes each with 2.5 mL HBsAg-positive serum mixed with 3.5 mL medium was prepared, and 30 μL Tween-80 was added to one of the tubes followed by mixing and 45 min incubation at RT. Thereafter, each mixture was added on the top of one gradient and covered with ~ 500 μl mineral oil, followed by centrifugation at 28,000 rpm in 10 **°**C for 8.5 h, using a SW28 rotor (Beckman Coulter). Mineral oil was removed thoroughly and 27 fractions were drawn from the top of the gradient (500 μl for the first 1–4 fractions and then 300 μl for the rest), and were kept at 4 **°**C on ice. Nucleic acid extraction was carried out as described above after dilution of 20 μL of each fraction with 180 μL dH_2_O. Then, 5 μL of extracted material were analysed by real-time PCR of HBV RNA and HBV DNA as described above.

### Reverse transcriptase PCR using a polyA-targeting reverse primer

In order to study the size and polyadenylation of HBV RNA in serum, samples from four HBeAg-positive patients were analysed by PCR analysis of cDNA that was created using a primer that binds to polyA sequences. The amplifications were designed to visualise full-length HBV RNA and the 3′ part of the HBV RNA, or to quantify two target regions (core and X) of the genome, as described in detail in the Additional file 1.

### Sequencing of amplicons of cDNA from 5′ and 3′ epsilon regions of HBV RNA

Observations in the present study and previously by others indicate that HBV RNA is present in virus like particles, and thus should have a functional encapsidation signal. There might still be mutations, that change the structure of the loop in epsilon segment where polymerase initiates reverse transcription. Therefore, we sequenced amplicons from a reverse transcription (RT) PCR comprising an RT step using a reverse primer (TTTTTTTTTTTTTTTTTGWAGCTC) that binds to the 3′ terminal poly-A tail, followed by PCR specific for the 5′ part of full-length RNA using primers 1819F (ACTTTTTCACCTCTGCCTAATCATC) and 1966R (TCAGAAGGCAAAAACGAGAGTAACT), and for the 3′ part using primers 1603F (GTTGCATGGAGACCACCGTGAAC) and 1882R (GCACAGCTTGGAGGCTTGA). The obtained amplicons were subjected to Sanger sequencing including cycle sequencing reaction using the same primers as in the PCR and automated reading of chain terminated fragments in an ABI 3130 XL Genetic Analyzer.

### Extraction of HBV nucleic acids from liver tissue

These procedures were performed as described previously [11]. Briefly, liver tissue pieces (≈ 5 mg), which had been stored in **–** 70 °C, were homogenized in a MagNA Lyser instrument (Roche Diagnostics). Nucleic acids were extracted in the MagNA Pure (Roche) instrument according to the manufacturer**’**s protocol.

### Digital PCR quantification of precore and pregenomic RNA

In order to study if the HBV RNA in serum was pregenomic RNA (as would be expected) or to some extent also precore RNA (which includes an additional ≈ 25 nt in the 5′ end) we quantified HBV RNA with PCR specific for either precore RNA or pregenomic and precore RNA by means of different forward primers targeting the 5′ end (HBV1803F, GCACCAGCACCATGCAACTT for precore, and HBV1825F, TCACCTCTGCCTAATCATCTCTTG, for pregenomic RNA. Discrimination of these RNA species was previously reported by Laras et al. using conventional PCR and quantitative estimates from gel electrophoresis [12]. We instead applied digital PCR, using HBV1966R as reverse primer and HBV1862–1886 as probe with a FAM fluorophore. The digital PCR technique has two advantages: It provides absolute copy numbers and is less sensitive to differences in PCR efficiency which might influence comparison based on so-called Ct (threshold cycle) values from real-time PCR.

These analyses provided one count for precore RNA and one for precore RNA + pgRNA; pgRNA was then calculated by subtraction (precore RNA+pgRNA) – precore RNA.

### Statistics

The correlations between HBV RNA in serum and HBV DNA in serum, as well as pgRNA in liver tissue were analysed by Spearman’s rho and by regression analysis. Genotype impact was analysed by comparing the HBV RNA/HBV DNA ratio between genotypes using Kruskall-Wallis (all genotypes) or Mann-Whitney U (genotype D vs. non-D) tests.

## Results

### General

As shown in Table 1 the study includes both HBeAg-positive and HBeAg-negative patients, with high and low HBV DNA levels, moderate to minimal inflammation and viral genotypes A-D.

**Table 1:** Clinical and virological information for the 95 patients with chronic HBV infection

	HBeAg+ (*n* = 32)	HBeAg– (*n* = 63)
Age (years, median, IQR)	27 (24–31)	34 (30–43)
Sex (M/F)	22/10	37/26
Genotypes (A/B/C/D)	4/7/9/12	13/9/3/38
HBV DNA (log copies/mL, median, IQR)	8.63 (7.73–9.42)	4.60 (3.94–5.79)
HBsAg (log IU/mL, median, IQR)	4.72 (4.14–5.24)	3.70 (3.14–4.05)
ALT/ULN (mean, SE)	1.47 (0.93–2.89)	0.84 (0.60–1.51)

*IQR* interquartile range, *SE* standard error

### HBV RNA in serum correlated with HBV DNA in serum and pgRNA in the liver

**Fig. 1:**
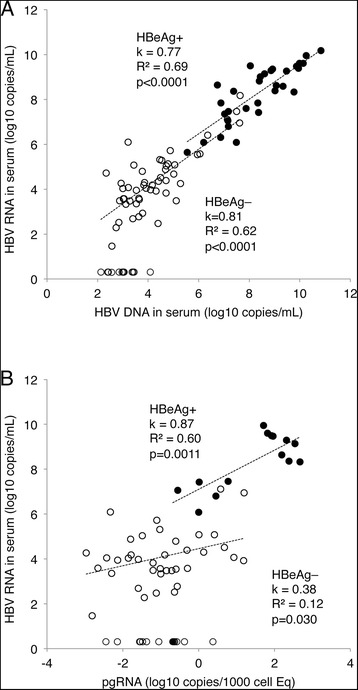
The HBV RNA level in serum correlated with HBV DNA in serum (**a**, *n* = 95), and with pgRNA (**b**, *n* = 65) in the liver. Filled circles, HBeAg+, unfilled circles HBeAg–

As shown in Fig. 1a, serum levels of HBV DNA and HBV RNA correlated with a Spearman’s rho of 0.93 and an overall R^2^ of 0.83, and with a similar slope for HBeAg positive (0.77) and negative (0.81) samples. In addition, the HBV RNA levels correlated with pgRNA in liver tissue, but with weaker correlations for HBeAg negative patients (Fig. 1b).

### HBV RNA in serum was present in particles with similar properties as HBV virions

In order to see if HBV RNA in serum was present within particles, amplification was performed with RNase digestion applied either directly to serum or after nucleic acid extraction in the MagNA Pure instrument (i.e. after lysis of viral envelopes and capsids). RNase treatment applied prior to nucleic acid extraction had no significant impact (the Ct value increased by 0.3 cycles), whereas RNase treatment after nucleic acid extraction removed almost all HBV RNA. These results suggest that there was essentially no free circulating HBV RNA in serum. A representative example of the effect of DNase and RNase treatment is shown in Table 2. The Ct value increased by 12 cycles indicating that the DNase and RNase degraded more than 99.9% of the HBV DNA and HBV RNA, respectively. This example, from an HBeAg-positive patient, also shows that the levels of HBV DNA and HBV RNA may be of the same magnitude.

**Table 2:** Cycle threshold (Ct) values obtained for one HBeAg-positive sample treated (or not treated) with DNase or RNase after nucleic acid extraction

	DNA amplification (PCR with DNA master mix and no RT step) Ct value	RNA amplification (RT-PCR with RNA master mix (incl reverse transcriptase and an initial RT step) Ct value
No enzymatic pretreatment	18.49	17.63
DNase	30.76	19.26
RNase	18.98	18.52
DNase and RNase	33.56	30.85

**Fig. 2:**
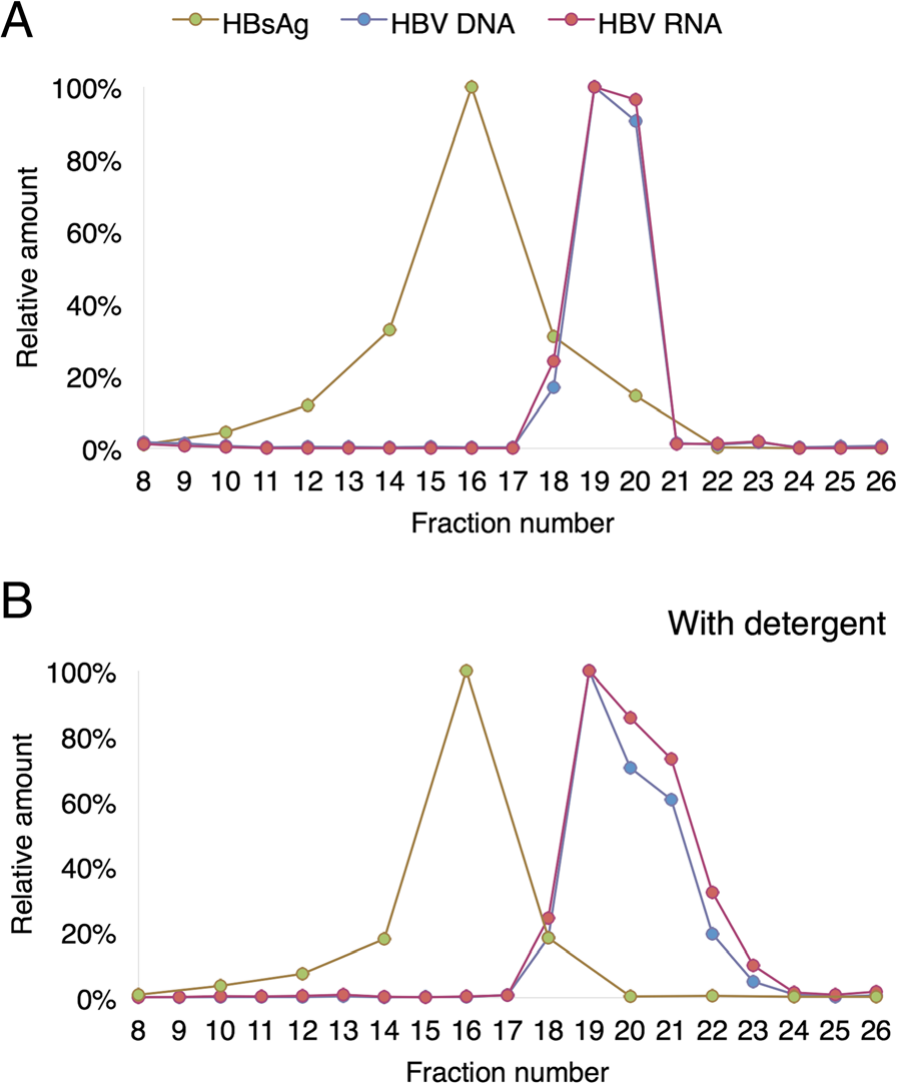
HBV RNA in serum was present in particles with similar properties as HBV virions. **a**) Quantification of HBV DNA, HBV RNA and HBsAg in Nycodenz gradient fractions from HBV positive serum. **b**) A parallel experiment in which the serum was treated with the detergent Tween-80 before fractionation

To further characterize the HBV RNA particles, a serum sample was fractionated on a Nycodenz gradient and analysed for viral DNA, RNA and HBsAg. As shown in Fig. 2, the fractions showed that HBsAg and viral DNA/RNA were well separated demonstrating that the gradient had enough resolution to separate viral particles from subviral particles in analogy with previous reports [13, 14]. The distribution of viral RNA in the fractions was almost identical to that of viral DNA (Fig. 2a), suggesting that the two different types of nucleic acids were indeed found in particles with similar densities. To verify that the DNA and RNA peaks corresponded to enveloped viral particles the serum sample was treated with detergent before centrifugation on the Nycodenz gradient. The detergent treatment led to a broadening of both the DNA and RNA peak towards higher Nycodenz concentration (Fig. 2b), probably reflecting the emergence of non-enveloped virus particles. In summary, the RNase and gradient experiments suggest that the viral RNA is associated with enveloped virus particles that are similar to the particles containing viral DNA.

### The HBV RNA was polyadenylated and of genomic length

After reverse transcription using a primer that targets polyA, different PCR strategies were applied. Primers designed to amplify the whole genome produced amplicons of the expected size (≈3100 bp), as shown in Additional file 1: Figure S1A-B. Amplification of the 3′ part of the HBV RNA showed weaker bands for the product that would be expected if the RNA was polyadenylated at nt 1807 (truncated form) rather than at nt 1933 (full-length form), as shown in Additional file 1: Figure S2. Real-time PCR of the cDNA using primers that target core and X regions showed similar levels of the two templates (Additional file 1: Figure S3).

### The lack of reverse transcription of HBV RNA was not explained by mutations in the 5′ epsilon sequence

**Fig. 3:**
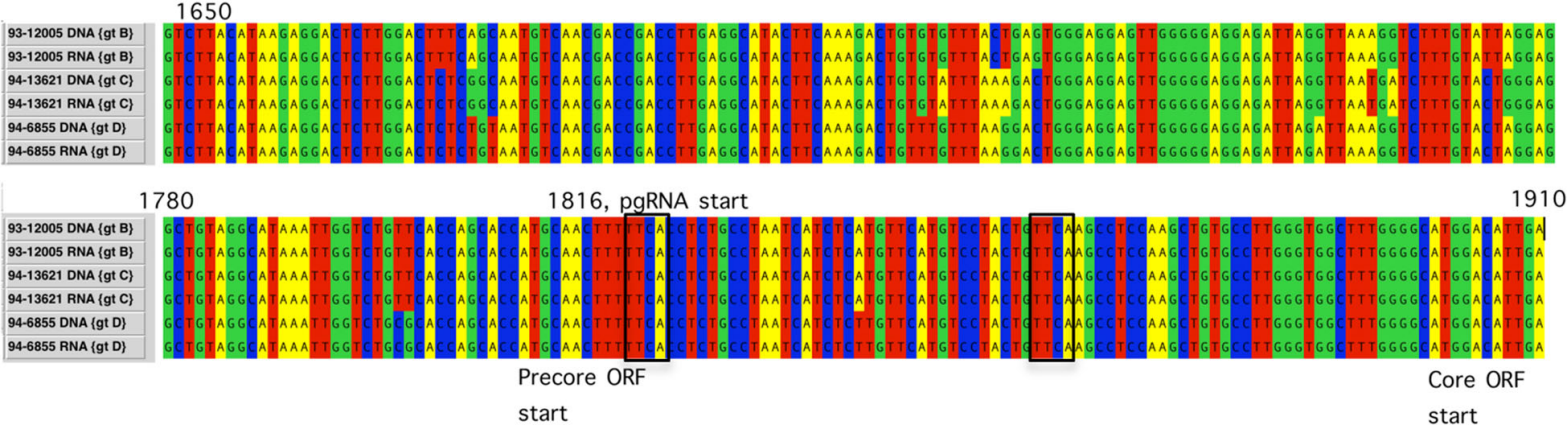
Sequences of the core promoter and precore regions of HBV identified in serum from three patients. The sequences were obtained by Sanger sequencing of products from separate amplification of DNA and RNA. The RNA sequences represent a merge of data from the 5′ end (amplified by primers 1819F and 1966R) and the 3′ end (amplified by primers 1603F and 1882R) of pgRNA. The DNA sequences were obtained from PCR using primers 1603F and 2058R. ORF, open reading frame. The priming (1863–1866 in the 5′ loop) and primer recipient sites (nt 1824–1827 in the 3′ loop) are boxed

The demonstration that HBV RNA was found in particles resembling infectious virus particles suggests that pgRNA was encapsidated, but not reverse transcribed. To investigate whether mutations that could be expected to affect reverse transcription were present in the epsilon (ε) sequence, the 5′ end of HBV RNA from three samples, representing genotypes B-D, was sequenced. No mutations predicted to influence the structure of the epsilon loop or priming of polymerase were observed (Fig. 3). The identification of the 5′ part of HBV RNA after reverse transcription with a reverse primer specific for the poly-A tail, indicates that the particle associated HBV RNA in serum represents the whole HBV genome.

### Nucleotide analogue treatment had little effect on the HBV RNA level

To further confim that the detection of HBV RNA was not due to unspecific amplification of HBV DNA we analysed a serum sample taken before and two months after the initiation of tenofovir therapy. During this time, the serum levels of HBV DNA declined from 6.98 to 4.23 log_10_ units, whereas HBV RNA levels declined from 5.12 to 4.66 log_10_ units.

### The ratio between HBV RNA and HBV DNA was genotype-related

**Fig. 4:**
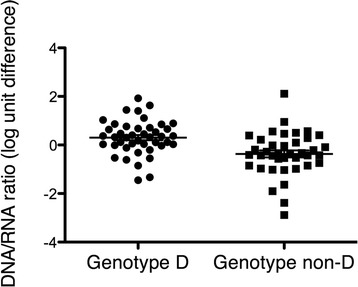
The ratio between HBV DNA and HBV RNA levels in serum was significantly higher in patients infected with HBV genotype D (*n* = 51) as compared with non-D genotypes (*n* = 44; *p* = 0.0001)

The ratio between HBV DNA and HBV RNA levels in serum was significantly associated with HBV genotype (*p* = 0.0009 by Kruskal-Wallis test). As shown in Fig. 4, a higher DNA/RNA ratio was observed in genotype D as compared with the other genotypes (median 2.10 vs. 0.45; *p* = 0.0001 by Mann-Whitney U test). The higher DNA/RNA ratio in genotype D was present in both HBeAg-positive (*p* = 0.01) and HBeAg-negative patients (*p* = 0.0007).

### Precore HBV RNA was present in serum

**Fig. 5:**
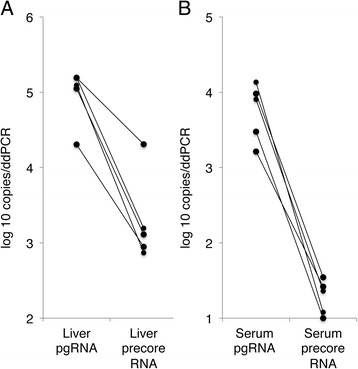
Levels of precore RNA and pgRNA in liver tissue (**a**) and serum (**b**) as measured by digital PCR. The level of pgRNA was on average 30 times higher (1.5 log_10_ units) than precore RNA in liver tissue, as compared with 200 times higher (2.3 log_10_ units) in serum, indicating that precore RNA is encapsidated and secreted to serum 6.6 times less effectively than pgRNA

In order to explore if the full-length HBV RNA in serum was pregenomic RNA (pgRNA) only, or if also precore RNA was present, we used two digital PCR assays, which discriminate between the two transcripts. These assays were applied on both serum and liver tissue samples from 5 patients. As shown in Fig. 5, the concentration of pgRNA in liver tissue was 30 times higher (1.5 log_10_ units) than of precore RNA. In serum, the levels of pgRNA were 200 times higher (2.3 log_10_ units) than precore RNA levels. The higher ratio in serum than in liver tissue corresponds with 6.6 times more efficient encapsidation of pgRNA than precore RNA.

## Discussion

In this study, we have analysed HBV RNA in serum from patients with chronic infection with the virus. A strong correlation between viral RNA and DNA was identified, and their relation was influenced by HBV genotype. Gradient fractionation and RNase treatment suggested that HBV RNA in serum was present in particles that resemble infectious particles carrying viral DNA. Sequencing of the 5′ epsilon part of the RNA did not reveal any mutations that would explain the lack of reverse transcription. Not only pgRNA, but also precore RNA was identified in the serum samples.

The serum levels of HBV RNA in our study were almost as high as HBV DNA levels. In previous studies the RNA levels have been between 0.8 and 2.8 logs lower than the DNA levels [6, 7, 15]. We believe that the quantitative relation between HBV RNA and HBV DNA observed in our study is accurate because we used the same extracted nucleic acid specimen for both analyses, which were performed in parallel using the same primers and probes for DNA and RNA. To ensure that the RNA assay did not amplify DNA we pre-treated the extracted nucleic acids with DNase and ran PCR assay in parallel without the reverse transcriptase enzyme. By doing so, we verified that the high HBV RNA levels were not due to amplification of HBV DNA (the DNase had > 99% efficiency). The strategy to use the same primers and probes and to run DNA and RNA amplifications in parallel probably contributed to the strong correlation between HBV DNA and HBV RNA levels with R^2^ of 0.82. This correlation was stronger than between HBV RNA in liver tissue and serum, suggesting that the secretion of viral particles containing HBV RNA into the blood is strongly linked to that of mature viral particles, and less representative of intracellular HBV RNA levels.

The experiments with RNase treatment and gradient centrifugation showed that HBV RNA was contained in particles with similar properties like that of infectious HBV particles, findings that support and extend previous observations in patients and in vitro. Like Jansen et al. [7], we found that RNase treatment before nucleic acid extraction did not affect the RNA levels detected. Wang et al. recently used sucrose gradient fractionation to show that HepAD38 cells treated with the nucleoside analogue entecavir secrete HBV RNA in particles with similar density as HBV virions [15]. Our results extend those observations by showing that HBV RNA particles with a similar density as HBV DNA particles were observed also in serum of infected individuals and also in the absence of treatment with nucleotide analogues.

The finding that HBV RNA was packed in capsids suggested that the epsilon (ε) sequence of pgRNA is functional, because this sequence is critical for encapsidation. There might still be mutations in the short loop that carries a signal to the polymerase to initiate reverse transcription [16]. In order to check this, we sequenced the 5′ epsilon part of HBV RNA from 3 patient serum samples (1 for each of HBV genotypes B, C and D) after creating cDNA with a reverse primer that targets poly-A in the 3′ end. The results indicated that the HBV RNA in these particles was full-length pgRNA, and the finding that cDNA could be synthesised by using a primer targeting poly-A indicates that RNA not at all had been digested by RNase H activity of the polymerase. No mutation was found in epsilon that would explain the lack of polymerase activity in these defective viral particles. Amplifications after reverse transcription with a primer that binds to polyA confirmed that the RNA was of genome length and that a truncated form with an alternative polyadenylation site constituted a minor fraction of the RNA. Real-time PCR quantification of the core and X regions showed similar concentration of the two templates, which further supported that the RNA was of genomic length.

The high levels of HBV RNA in serum seem to reflect that reverse transcription often fails, resulting in the secretion of RNA containing viral particles. Our findings indicate that this failure rate might be influenced by HBV genotype. The ratio between HBV DNA and HBV RNA was higher in genotype D suggesting that genotype D viruses might have a more effective reverse transcription. Reverse transcription of HBV RNA to DNA comprises a priming at a bulge of the 5′ epsilon loop [16], followed by translocation of the primed DNA to a receptor sequence located in the 3′ part of pgRNA, which has a complex secondary structure [17]. Sequence differences in this secondary structure might influence the efficiency of the primer translocation in a manner that results in genotype related differences in the DNA/RNA ratio.

After identifying that the HBV RNA in serum was likely of full length, we used digital PCR to analyse the quantitative relationship between the two full-length RNA species, precore RNA and pgRNA. This was achieved by using two different forward primers, one specific for precore RNA and one targeting both precore and pregenomic RNA, as previously shown by Laras et al. [12]. By using digital PCR for this analysis rather than real-time PCR we reduced the risk of bias from differences in amplification efficiency that might be caused by using different forward primers. By analysing both liver tissue and serum from the same patients we could also study if a difference of the proportions of these HBV RNA species in serum was due to selective encapsidation of either of them. In serum the concentration of pgRNA was approximately 300 times higher than precore RNA, which is consistent with a previous report that concluded that precore RNA represented less than 1% of the total pgRNA and precore RNA in serum [7]. This mainly seems to be the result of relatively less transcription of precore RNA, because in liver tissue the concentration of pgRNA was approximately 30 times higher than precore RNA. Comparison of serum and liver tissue ratios suggests that encapsidation and secretion of precore RNA indeed occurs, although this process appears to be almost seven times less efficient than for pgRNA.

## Conclusions

In summary, we detected high levels of HBV RNA in serum from patients with chronic HBV infection. These levels seem to reflect production of high amounts of defective viral particles containing full-length pgRNA that of some reason are not reverse transcribed into DNA. Additional studies are required to clarify if this is due to the absence of polymerase in these particles or has other explanations.

## Additional file

Additional file 1: **Figures S1-S3.** In order to study the size and polyadenylation of HBV RNA in serum, samples from four HBeAg-positive patients was analysed by different PCR strategies. (DOCX 1019 kb)

## Abbreviations

HBsAg: Hepatitis B surface antigen
HBV: Hepatitis B virus
HCC: Hepatocellular carcinoma
pgRNA: pregenomic RNA
