# Supplementary material for: High serum levels of pregenomic RNA reflect frequently failing reverse transcription in hepatitis B virus particles
Source: Virol J. 2018 May 15;15:86. doi: 10.1186/s12985-018-0994-7 (PMC5952638; doi:10.1186/s12985-018-0994-7)

Supplementary Figures 1-3.

In order to study the size and polyadenylation of HBV RNA in serum, samples from four HBeAg-positive patients was analysed by different PCR strategies.

Supplementary Figure 1A-B.

First, cDNA was created using random primers (lanes 1-4), or poly-A targeting primers with a 3’ end designed to preferentially bind to truncated HBV RNA (GAGACTCGACTCCA-CAACCATTTTTTTTTTTTTTTTTGTGAACAGAC, lanes 5-8) or to full-length HBV RNA (lanes 9-12, ACCACGCTATCGCTACTCACTTTTTTTTTTTTTTTTTGTAGCTC).

Then PCR was performed using primers designed to amplify the whole genome.

A) Agarose gel after the first PCR: 1821F, TCTTTTTCACCTCTGCCTAATCA and 1825R, GAAAAAGTTGCATGGTGCTGGT. Weak bands of ≈ 3200 nt size are visible in lanes 10-12.

B) The products from the first PCR were subjected to nested PCR using 1925F, GAGCTTCTGTGGAGTTACTCTC and 1801R, CAGACCAATTTATGCCTACAGCCT. The agarose gel shows the products from lanes 5-8 in 1A. The first lane is negative, lanes 2-3 shows a product of ≈ 3100 bp size, and lane 4 two bands of approximately 1200 and 800 bp size which probably is the result alternative annealing of one or both of the inner primers.


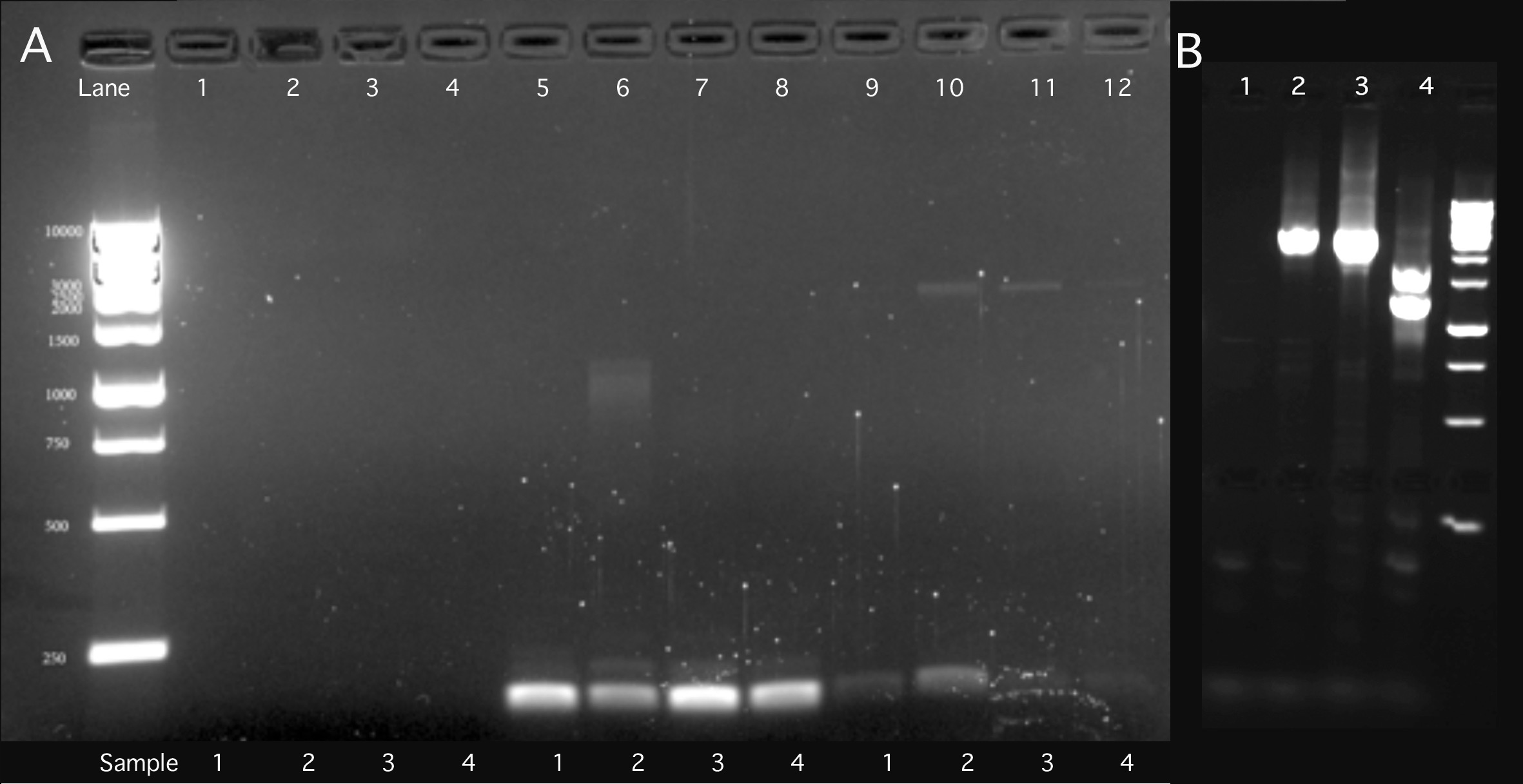


Supplementary Figure 2. Amplification of the 3’ part of the HBV RNA, using 1550F, CGTCTGTGCCTTCTCATCTG, as forward primer, combined with a reverse primer that was specific for a tag sequence that was introduced in the reverse transcription step (ACCACGCTATCGCTACTCAC). The results show weaker bands for the product that would be expected if the RNA was polyadenylated at nt 1807 (truncated form, ≈300 bp band) rather than at nt 1933 (full-length form, ≈430 bp band).

Supplementary Figure 3. After an RT step using a reverse primer that targets polyA, real-time PCR that quantify core (nt 2367-2454) and X (nt 1550-1627) regions was performed. Similar concentration of the two templates were observed in serum samples from four patients. The results agree with the interpretation that HBV RNA in serum represents full-length RNA.


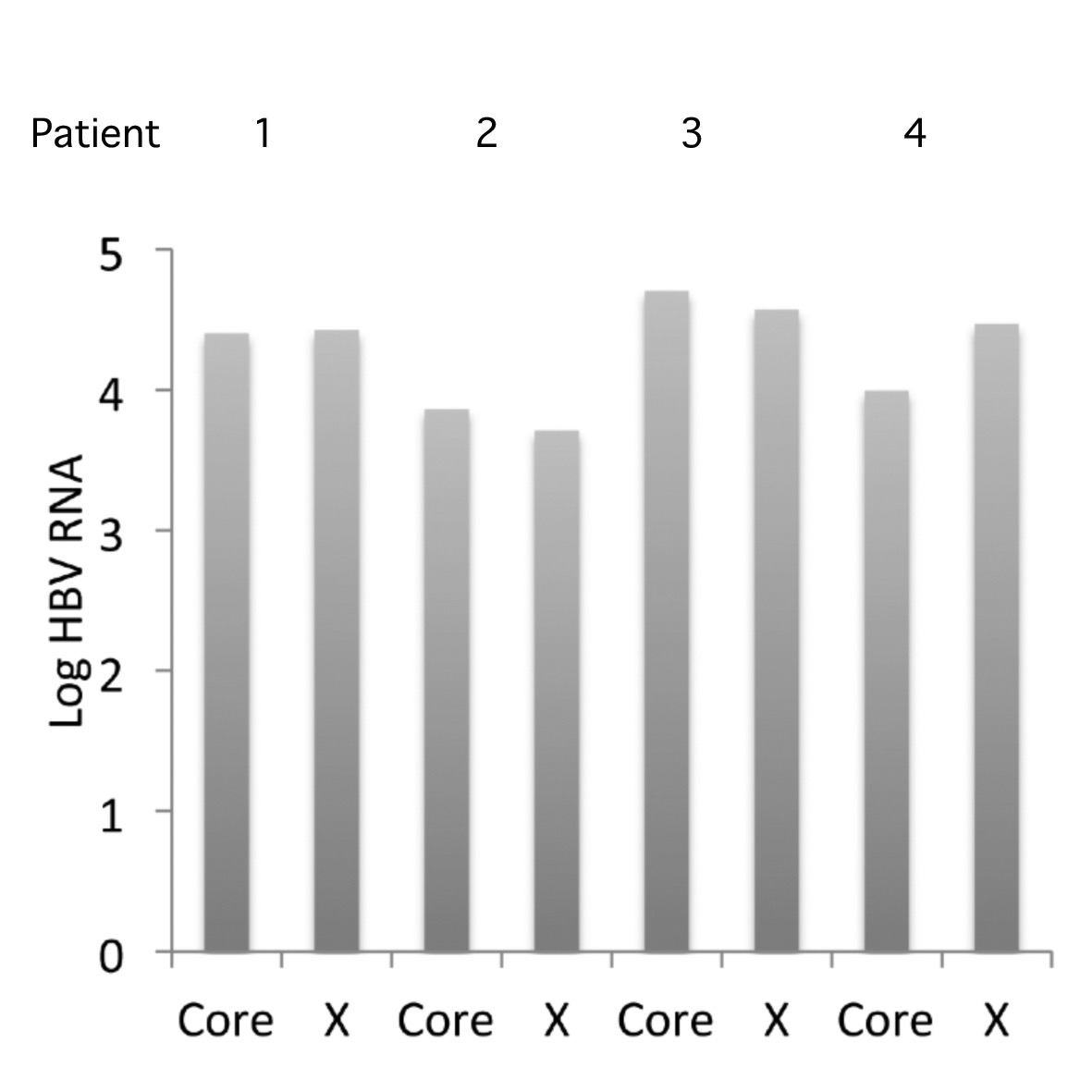

Supplement: Supplementary file 1 — Figures S1-S3. In order to study the size and polyadenylation of HBV RNA in serum, samples from four HBeAg-positive patients was analysed by different PCR strategies. (DOCX 1019 kb) [file 12985_2018_994_MOESM1_ESM.docx]
